# Using combined Global Position System and accelerometer data points to examine how built environments and gentrification are associated with physical activity in four Canadian cities

**DOI:** 10.1186/s12966-022-01306-z

**Published:** 2022-07-07

**Authors:** Caislin L. Firth, Yan Kestens, Meghan Winters, Kevin Stanley, Scott Bell, Benoit Thierry, Kole Phillips, Zoé Poirier-Stephens, Daniel Fuller

**Affiliations:** 1 University of Washington, 1959 NE Pacific St, Seattle, WA 98195 United States; 2 Université de Montréal/Centre de Recherche du CHUM, Pavillon S, 850 rue St-Denis, Montréal, QC H2X 0A9 Canada; 3 Faculty of Health Sciences, Simon Fraser University, 8888 University Drive, Burnaby, BC V5A 1S6 Canada; 4 University of Saskatchewan, 105 Administration Place, Saskatoon, S7N 5A2 Canada; 5 Memorial University of Newfoundland, 230 Elizabeth Avenue, St. John’s Newfoundland, A1C 5S7 Canada

**Keywords:** Physical activity, Global positioning systems, Accelerometry, Walkability, Urban sprawl, Gentrification

## Abstract

**Background:**

Built and social environments are associated with physical activity. Global Positioning Systems (GPS) and accelerometer data can capture how people move through their environments and provide promising tools to better understand associations between environmental characteristics and physical activity. The purpose of this study is to examine the associations between GPS-derived exposure to built environment and gentrification characteristics and accelerometer-measured physical activity in a sample of adults across four cities.

**Methods:**

We used wave 1 data from the Interventions, Research, and Action in Cities Team, a cohort of adults living in the Canadian cities of Victoria, Vancouver, Saskatoon, and Montreal. A subsample of participants wore a SenseDoc device for 10 days during May 2017–January 2019 to record GPS and accelerometry data. Two physical activity outcomes were derived from SenseDoc data: time spent in light, moderate, and vigorous physical activity; and time spent in moderate or vigorous physical activity. Using corresponding GPS coordinates, we summarized physical activity outcomes by dissemination area—a Canadian census geography that represents areas where 400 to 700 people live- and joined to built (active living space, proximity to amenities, and urban compactness) and gentrification measures. We examined the associations between environmental measures and physical activity outcomes using multi-level negative binomial regression models that were stratified by city and adjusted for covariates (weekday/weekend), home dissemination area, precipitation, temperature) and participant-level characteristics obtained from a survey (age, gender, income, race).

**Results:**

We found that adults spent more time being physically active near their homes, and in environments that were more walkable and near parks and less time in urban compact areas, regardless of where participants lived. Our analysis also highlighted how proximity to different amenities was linked to physical activity across different cities.

**Conclusions:**

Our study provides insights into how built environment and gentrification characteristics are associated with the amount of time adults spend being physically active in four Canadian cities. These findings enhance our understanding of the influence that environments have on physical activity over time and space, and can support policies to increase physical activity.

**Supplementary Information:**

The online version contains supplementary material available at 10.1186/s12966-022-01306-z.

## Background

Physical, social, and economic environments [1] have both positive and negative consequences on health outcomes. One of the most apparent impacts is on physical activity and how environments influence when and where people choose to be active. Creating models that accurately capture how individuals interact with their cities and how exposure to urban environments can influence physical activity behaviour is an important public health objective. There is evidence that active living environments [2], urban compactness [3], and public transit [4] (i.e., urban environmental exposures), and gentrification [5] (i.e., social environmental exposures) are positively associated with physical activity. Review studies and conceptual papers suggest new measurements using Global Positioning Systems (GPS), accelerometers, and related sensors that capture where, when, and how active people are in their communities can enrich our understanding of the associations between urban environmental exposures and physical activity [6]. With advances in Geographic Information Systems (GIS) and spatial data analysis, researchers are developing better methods to operationalize concepts like active living environments and urban compactness [7]. However, these technologies and our ability to deploy them at a large scale have created new challenges to our understanding. For example, simply defining an exposure is an increasingly challenging task [8]. In addition, selective daily mobility bias, people selecting to go to places that support the behaviours they want to engage in, may hamper the ability to make causal claims about exposure outcome relationships with combined GPS and accelerometer data [9, 10].

Despite the promise of combining GPS and accelerometer data for healthy cities research, there are still relatively few studies that have done so. In a systematic review, 79 published articles were identified that combine GPS and accelerometer data to examine physical activity patterns, and these tended to study specific populations, have small samples, and limited scope in terms of exposures and geographies. For example, almost half of these studies focused on children (45/97) and the median sample size was 148 participants [11]. Forty percent of studies used only a single built environment exposure and few included multiple cities within a single study. Findings consistently show that home location, walkability, greenspace (including parks), and urban compactness are associated with physical activity [12, 13]. Topics such as the impact of social and environmental factors including gentrification and proximity to primary and secondary education or hospitals relate to physical activity are more poorly covered. For example, a study including 223 participants from 5 different states (California, New Mexico, North Carolina, Ohio, and Pennsylvania) showed that homes and roads accounted for 40% of physical activity, while parks were used for 13% of Moderate or Vigorous Physical Activity (MVPA) [14]. The study also showed differences in how people used spaces in California compared to other states. Studies rarely use comparable metrics for physical activity outcomes, or exposure definitions, and there is great diversity in the modelling approaches in these types of studies [11]. In a systematic review of activity space in studies of the environment and physical activity, 47 studies were included with the majority of studies examining the size/shape of the activity space (*n* = 20) or for specific routes (*n* = 12) with all studies aggregating data to the trip, day, or multi-day level [15]. There is a need for more multi-city studies examining associations between built and social environment exposures and physical activity using GPS and accelerometer methods at a temporal unit of analysis.

The purpose of this study was to examine the association between GPS-derived exposure to built environment and gentrification characteristics and accelerometer-measured physical activity in a sample of adults across Canadian four cities. We use high spatial resolution exposures and an analytic approach that goes beyond current methods, that rely on static environmental exposures, to assess exposure to characteristics by using accelerometer data to document ‘where’ people spend their time and ‘dose’ of how much time they spend in different environments. Consistent with existing literature, we hypothesize that being near home will be associated with physical activity, also that areas with higher walkability, greenspace (including parks), and more urban compactness will be associated with physical activity.

## Methods

### Study design

This study uses the wave 1 data from the Interventions, Research, and Action in Cities Team (INTERACT) study [16]. INTERACT is a cohort study that is designed to examine using natural experiments the impact of transportation interventions in four Canadian cities (Victoria, Vancouver, Saskatoon, and Montreal) [17]. To provide context, we have included a socio-demographic data for four different cities included in our study (Additional file 1: Supplement A).

### Study participants

Recruitment for wave 1 occurred from May 19—October 21, 2017 (156 days) in Victoria; April 20 – September 20, 2018 (123 days) in Vancouver; September 19, 2018 – January 4, 2019 (108 days) in Saskatoon; and June 6—December 21, 2018 (199 days) in Montreal. Inclusion criteria across all sites were being at least 18 years old, being able to read or write English (or French in Montreal) well enough to answer an online questionnaire, and not planning to move out of the city in the next two years. Site-specific inclusion criteria involved living in the Capital Regional District and cycling at least once a month in the city of Victoria; living within 3 km of the Greenway in Vancouver; riding the bus at least once in a typical month, or living within 800 m of the proposed BRT Bus Rapid Transit) lines in Saskatoon; and living on the Island of Montreal, Laval, or the South Shore in Montreal. The minimum requirement for participation was completing an online survey that measured health, physical activity, social participation, travel behaviour, and socio-demographic characteristics using validated measures. Participants could also choose to wear a SenseDoc [18] device for 10 days during waking hours, which recorded GPS and accelerometry data. The location data in the SenseDoc is measured using GPS at 1 Hz and accelerometer measuring at 50 Hz continuously, as long as the device was charged and on. The participants analyzed in this paper were those who completed the health survey and wore the SenseDoc device.

### Measures

#### Outcomes

We examined two physical activity outcomes: total time spent in light, moderate, and vigorous physical activity (PA); and time spent in moderate or vigorous physical activity (MVPA). Minutes of sedentary, light, moderate, and vigorous physical activity were calculated using accelerometer data collected by the SenseDoc device worn by participants for 10 days. Minute by minute location-based physical activity level was calculated using methods applied in past research [16]. First, raw accelerometer data was converted to counts using published methods (implemented with Python code) [19, 20]. Vertical axis counts were then used for wear detection. The Choi algorithm was used to calculate device wear and non-wear [21]. For times when the accelerometer was worn, Troiano’s physical activity cut points were used to classify sedentary, light, moderate, and vigorous physical activity at the minute level [22]. GPS at the second level location data were joined to the accelerometer data at the second level. The median location at each minute was taken as the location in order to aggregate the GPS data to the minute level. We did not apply wear time criteria (e.g., 10 h of valid data) to the physical activity data as our objective was to keep as much of the data as possible for all participants.

In order to link physical activity outcome data with built environment and gentrification characteristics, the daily sum of minutes spent in either, sedentary, light, moderate, or vigorous physical activity in each dissemination area (DA) for each participant for each day was computed. Dissemination Areas Canadian census geographies representing small areas with an average population of 400 to 700 people [23].

#### Neighborhood built environment and gentrification exposures 

We examined how neighborhood environment exposures were linked to the amount of total physical activity (light, moderate, and vigorous) and moderate or vigorous physical activity. Our environment indicators were either measured at the DA level, or census tract (CT) level (CTs are stable areas made up of multiple DAs with populations of 2,500 to 8,000 people (average of 4,000)) [24]. Exposure to different environments (e.g., active living environments, gentrification, proximity to amenities, and urban compactness) are described in the following paragraphs. We then joined our physical activity dataset to each of the environmental datasets at either the DA or CT level to create our final dataset.

##### *Active living space exposure*

Our active living exposure was measured from the 2016 Canadian Active Living Environments (Can-ALE) database. This geographic-based set of measures is intended to capture the active living friendliness of Canadian communities. A Can-ALE score is calculated by counting the number of intersections, dwellings, points of interest, and public transit stops within a circular 1-km buffer from the DA centroid. In Can-ALE, the four measures are then transformed into a Z-Score, combined into a composite measure, and divided into quintiles representing the favourability of the active living environment within each DA from 1 (very low) to 5 (very high). For example, in areas within the least amount of active living there are an average of 12 points of interest within a 1-km buffer compared to 429 points of interest in areas of very high active living.

##### *Proximity to amenities measures*

The proximity measures database released by Statistics Canada in April 2020 provides the proximity to 10 amenities types at the dissemination block-level [25]. Dissemination blocks cover all of Canada and are equivalent to a city block bounded by intersecting streets and are nested within dissemination areas. Two of the proximity measures are based on driving distance: proximity to employment within a 10 km buffer of the dissemination block centroid and proximity to healthcare within a 3 km buffer. The remaining 8 measures rely on walking distance: closeness to grocery stores, pharmacies, public transit, and neighbourhood parks in a 1 km buffer of the dissemination block centroid and proximity to primary, and secondary education, childcare, and libraries within a 1.5 km walking buffer of the dissemination block centroid. Each proximity measure has been normalized on a 0 to 1 scale where 0 indicates the lowest proximity and 1 the highest proximity in the data. We aggregated each proximity measure and calculated the median value for each DA. For analysis, we created quintiles of each proximity measure across the four cities so that a one-unit change corresponds with a one-quintile difference in proximity. We assumed the relationship between quintile measures and physical activity outcomes functioned in a linear fashion; such that, effect sizes represent the average effect across all 1-unit differences in a quintile measure.

##### *Urban compactness*

Which can be thought of as the inverse of urban sprawl, was calculated using nine urban form indicators representing four dimensions; density, mix use, street connectivity, and centering are used in the index construction [26]. The indicator was developed at the CT level in Canada using Bayesian multivariate spatial factor analysis. The urban compactness is similar to one developed by Ewing et al. in the United States [13].

##### *Gentrification*

The GENUINE database of Canadian gentrification measures are calculated from census data at the CT level in all Canadian metropolitan areas [27]. The measures rely on different combinations of change in census measures related to income, housing, occupation, education, and age. We used the measure adapted from Ding et al. to classify areas that had experienced gentrification during 2006 to 2016. A census tract was ‘gentrifiable’ or eligible to gentrify in 2006 if the median household income was below that of the respective metropolitan area. A gentrifiable census tract was classified as ‘gentrified’ by 2016 if: a) the median gross rent or median home value increased more than citywide increases and b) the proportion of college-educated residents increased more than citywide increases [28, 29]. We used a 3-level gentrification measures to represent areas that are ‘high socioeconomic status (SES) tracts, not eligible to gentrify,’ ‘low SES tracts, did not gentrify,’ and ‘gentrified tracts.’ We joined the CT gentrification measure to their corresponding DA.

#### Covariates

We included both demographic and weather covariates. Participant demographics were provided through survey data (age, gender, race). In regression models, we used four age groups (18–24, 25–44, 45–64, 65 + years), three gender groups (male, female, trans/non-binary/other), three income groups (annual household income < $50,000, $50,000-$99,999, $100,000 +), and a broad race grouping (persons who identified as white or Caucasian, persons who identified as a visible minority or Indigenous). We included a day of the week indicator variable (weekend, weekday) for when the PA occurred. We also included a dichotomous variable to represent the dissemination area where the participants home address was location. We define this as the Home DA in the analyses. Finally, weather variables including the total amount of precipitation that day (mm), and the average temperature (in Celsius) were included.

#### Analyses

Analyses were conducted in R version 4.1.0 and RStudio version 1.4.1106 and StataSE 16. The GPS and accelerometer data were spatially joined to the exposure data using the *sf* package and the *st_join* function in R. Joined GPS and accelerometer data were aggregated by individual ID, date, and dissemination area so that total minutes, minutes of physical activity, and minutes of MVPA were calculated for each DA, for each day, for each person. Following this, all exposure measures were joined to the GPS and accelerometer data using the unique identifier for each dissemination area within the study area.

We conducted descriptive statistics to characterize our study sample by city. We mapped time spent in light, moderate, and vigorous physical activity (PA) as the sum of minutes spent in each DA across all observation days and participants, and present the outcomes as quintiles to highlight spatial patterns in physical activity across cities [30].

We examined the associations between built environment and gentrification characteristics and physical activity outcomes in all four cities. To answer our research questions, we fit a series of multi-level negative binomial regression models to examine the links between the amount of time spent being physically active with built environment and gentrification characteristics. Models were stratified by city to answer whether environmental correlates of physical activity are consistent across place. Separate models were fit for our two outcomes, PA and MVPA. Each model adjusted for participant demographic (age, gender, income, race group) and covariates (weekend, home dissemination area, precipitation, temperature). To account for correlation within repeated measures for individuals over time, we included a random intercept by person and random slope as time (number of observation days). We present model coefficients as incidence rate ratios that can be interpreted as the average amount of time (in minutes) of either PA or MVPA that is associated with a particular level of a covariate relative to the reference level.

To examine the robustness of our study findings, we repeated the series of PA and MVPA models for DAs where participants spent at least 5 min per day. In doing so we were able to understand whether participants were just traveling through, or spending less than 5 min in a dissemination area, or spending more substantial amounts of time being physically active in a DA. All analysis code is available online [30].

The initial dataset included 2,493,887 min of matched accelerometer and GPS data for 544 participants. Once the data were aggregated at the person, day, and DA level, the complete dataset included 177,104 observations. The observations represented the number of minutes each participant spent per day in each DA they entered. We removed 2,157 records where geographic location was missing or date was missing. In addition, we removed 861 observations, associated with 4 participants because of device error.

## Results

Table 1 presents the participation and demographic characteristics of the 544 adult participants stratified by city. Of the 1155, 316, 334, and 281 participants who completed the health survey in Montreal, Saskatoon, Vancouver, and Victoria, 159, 85, 150, and 160 wore a SenseDoc device. Of those participants 157, 78, 150, and 152 participants were included in the analyses in Montreal, Saskatoon, Vancouver, and Victoria, respectively. The demographic results show that participants tended to be women and white; half of the participants had a household income greater than $100,000, similar to the overall cohort of participants who completed the health survey [16]. Participants recorded between 5.7 and 9.7 h per day of accelerometer data, the median duration in each city. The average minutes spent within a DA in each city was Victoria (19 min, range = 1 min to 3h46min), Vancouver (8 min, range = 1 min to 4h28min), Saskatoon (27 min, range = 1 min to 5h23min), Montreal (10 min, range = 8h2min).

**Table 1:** Demographic characteristics of participants by city

	**Montreal**^a^		**Saskatoon**		**Vancouver**		**Victoria**	
Data collection period	July 10th 2018-Feb 10th 2019		Oct 2nd 2018-Feb 21th 2019		May 2nd 2018-Dec 16th 2018		May 31st 2017-Dec 3rd 2017	
Participant n (Health Survey)	1155		316		334		281	
Participants n (SenseDoc)	159		85		150		160	
Participants n (Analysis)	157		78		150		152	
	**Median (25**^**th**^** -75**^**th**^** percentile)**							
Count of accelerometer tracking days	11	(10–11)	10	(9–11)	11	(10–11)	11	(10–11)
Hours of recording/day	5.3	(2.5–9.0)	9.7	(5.3–13.2)	5.7	(2.8–9.4)	8.1	(4.4–11.7)
Age group	**n (percent)**							
18–24 years	7	4%	9	11%	1	1%	6	4%
25–44 years	82	52%	50	59%	16	11%	76	50%
45–64 years	56	36%	17	21%	74	49%	56	37%
65 + years	12	8%	2	2%	59	39%	15	10%
Missing	0	0%	7	8%	0	0%	0	0%
Gender								
Woman	95	61%	60	71%	101	67%	77	51%
Man	60	38%	24	28%	49	33%	72	47%
Transgender/Non-binary	2	1%	1	1%	0	0%	3	2%
Race^b^								
White	146	93%	66	78%	130	87%	139	91%
Black	2	1%	3	4%	1	1%	0	0%
Indigenous	0	0%	3	4%	1	1%	2	1%
Latinx/Latin American	4	3%	3	4%	5	3%	1	1%
West Asian	0	0%	1	1%	0	0%	0	0%
Asian	10	6%	8	9%	10	7%	11	7%
Other	3	2%	3	4%	7	5%	0	0%
Household income								
< $50,000	26	17%	37	44%	22	15%	25	16%
$50,000- $99,999	52	33%	19	22%	36	24%	55	36%
$100,000 +	79	50%	29	34%	92	61%	72	47%

^a ^Montreal recruited potential Sensedoc participants at random from the Health Survey because we did not have sufficient devices to allow any interested participant to use a Sensedoc

^b ^Racial categories are not mutually exclusive, participants can identify with more than one racial group. In addition, race or ethnicity were not asked consistently across study sites. We created 'West Asian' group from people who identified as Middle Eastern in Victoria or Vancouver and West Asian or Arab in Saskatoon or Montreal. The 'Asian' racial group includes people who identified as Asian in Victoria or Vancouver and Chinese, South Asian, South East Asian, Filipino, Korean, or Japanese in Saskatoon or Montreal. In multivariable analysis, race was a binary variable (white or Caucasian, Visible Minority and/or Indigenous)

**Fig. 1:**
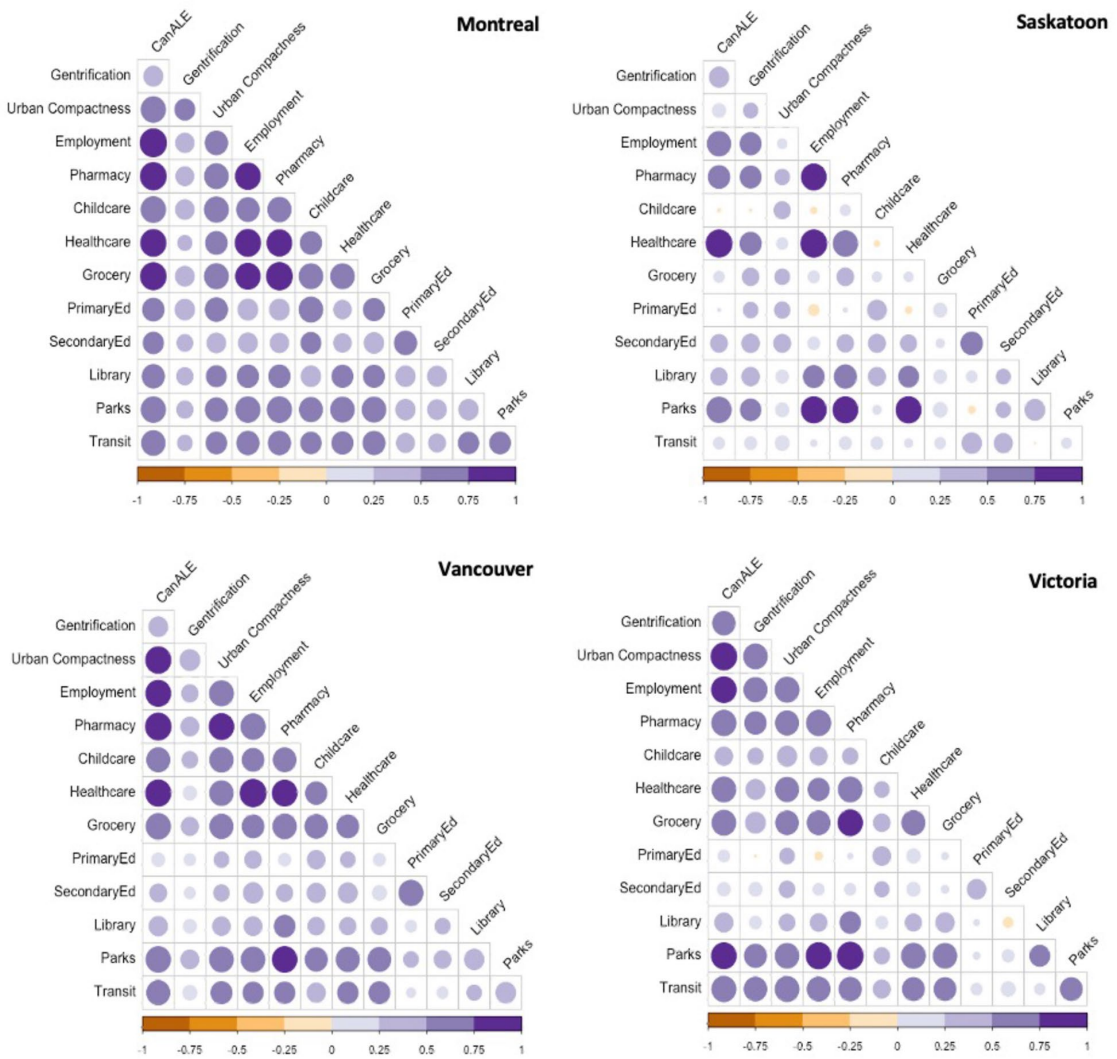
Correlations between built environment and gentrification characteristics by city

Correlations between the built environment and gentrification characteristics were different between the four cities. Notably, Can-ALE was highly correlated with numerous proximity measures including, employment, pharmacies, healthcare, and groceries. Across cities, not all correlations were positive. Saskatoon and Victoria showed patterns of weak negative correlations between social and built environment characteristics that did not appear in Montreal and Vancouver, including areas with greater proximity to employment and childcare in Saskatoon, or greater proximity to grocery stores and schools in Victoria Fig. 1.

### Associations with total physical activity

Results for total minutes of light, moderate, and vigorous physical activity show that across all four cities higher scores for CAN-ALE were associated with increased physical activity (Table 2). With each quintile increase in CAN-ALE the number of minutes participants spent being active increased by 15% more minutes per dissemination area each day (Vancouver) to 34% more minutes (Victoria). Yet, in three of the cities–Montreal, Vancouver, Victoria–proximity to public transit was negatively associated with time spent being physically active; the association between total minutes of physical activity and proximity to public transit in Saskatoon was not statistically significant. Other clear patterns in the data included urban compactness, which was associated with less physical activity across all four cities.

**Table 2:** Associations between built environment and gentrification variables and physical activity, by city^a^

	**Montreal**	**Saskatoon**	**Vancouver**	**Victoria**
**Number of people**	**157**	**78**	**150**	**152**
**Can-ALE (quintiles)**	**1.18 (1.13–1.23)**	**1.3 (1.15–1.47)**	**1.15 (1.11–1.19)**	**1.34 (1.29–1.39)**
**Gentrification (Ding)**				
High SES	Reference	Reference	Reference	Reference
Low SES	**0.89 (0.84–0.95)**	**0.44 (0.38–0.51)**	**1.15 (1.08–1.22)**	**0.8 (0.75–0.86)**
Gentrified	**0.93 (0.88–0.99)**	1.03 (0.87–1.21)	**1.15 (1.09–1.22)**	0.95 (0.88–1.02)
**Urban Compactness**	**0.66 (0.63–0.7)**	**0.79 (0.66–0.96)**	**0.51 (0.48–0.55)**	**0.56 (0.51–0.61)**
**Proximity (quintiles):**				
Employment	1.01 (0.98–1.05)	**0.82 (0.71–0.95)**	**0.9 (0.86–0.93)**	**0.81 (0.76–0.85)**
Pharmacy	**1.15 (1.11–1.19)**	**0.91 (0.83–0.99)**	**1.06 (1.02–1.09)**	**1.2 (1.16–1.24)**
Childcare	**1.05 (1.02–1.09)**	**1.09 (1.01–1.17)**	**0.83 (0.81–0.86)**	**0.88 (0.85–0.9)**
Health	0.98 (0.96–1.01)	**0.74 (0.67–0.83)**	**1.14 (1.1–1.19)**	**0.97 (0.93–1)**
Grocery	**0.93 (0.9–0.95)**	**0.79 (0.74–0.84)**	**1.07 (1.04–1.1)**	**1.06 (1.03–1.08)**
Primary education	**0.9 (0.88–0.92)**	**0.72 (0.68–0.76)**	**0.93 (0.9–0.95)**	**1.1 (1.07–1.12)**
Secondary education	**0.95 (0.93–0.96)**	**0.91 (0.86–0.95)**	**0.92 (0.89–0.94)**	**0.83 (0.82–0.85)**
Library	0.99 (0.98–1.01)	**1.15 (1.09–1.21)**	**1.12 (1.1–1.14)**	**1.08 (1.06–1.1)**
Transit	**0.89 (0.87–0.92)**	1 (0.92–1.08)	**0.85 (0.83–0.88)**	**0.93 (0.9–0.96)**
Parks	**1.07 (1.05–1.09)**	**1.29 (1.22–1.36)**	**1.12 (1.1–1.15)**	**0.96 (0.94–0.98)**
**Gender**				
Woman	Reference	Reference	Reference	Reference
Man	1.06 (0.85–1.33)	0.87 (0.61–1.25)	**0.7 (0.56–0.88)**	1.01 (0.83–1.22)
Non-binary	0.73 (0.28–1.87)	**15.12 (3.51–65.14)**		1.88 (0.95–3.73)
**Income groups**				
< $50,000	Reference	Reference	Reference	Reference
$50,000-$99,999	0.94 (0.68–1.31)	1.1 (0.72–1.68)	0.74 (0.53–1.03)	0.77 (0.58–1.01)
$100,000 +	0.86 (0.63–1.16)	**1.56 (1.08–2.26)**	**0.73 (0.54–0.99)**	**0.68 (0.52–0.89)**
**Race**				
White	Reference	Reference	Reference	Reference
Visible minority or Indigenous	1.03 (0.67–1.58)	0.75 (0.5–1.14)	0.96 (0.71–1.29)	0.94 (0.67–1.32)
**Age**	1.04 (0.89–1.22)	1.06 (0.82–1.36)	0.92 (0.79–1.07)	0.97 (0.85–1.11)
**Home DA**	**34.38 (31.26–37.8)**	**27.32 (21.88–34.1)**	**38.36 (34.44–42.72)**	**33.78 (30.85–36.99)**
**Weekend**	**0.95 (0.91–0.99)**	1.07 (0.97–1.17)	1.04 (1–1.09)	0.97 (0.93–1.01)
**Precipitation (mm)**	1 (0.99–1)	0.97 (0.93–1.02)	1 (0.99–1.01)	1 (1–1.01)
**Temperature (C)**	**0.99 (0.98–0.99)**	1 (0.99–1.01)	**1.02 (1.01–1.03)**	0.99 (0.98–1.01)

^a ^Results for each model coefficient are reported as incidence rate ratios and 95% confidence intervals

Bold results indicate statistically significant results (*p*-value < 0.05)

When we mapped time spent in physical activity, areas in the top quintile of time spent tended to be outside of the downtown core in each city [30]. Participants spent less time being physically active in low socio-economic neighbourhoods that had not gentrified and the patterns within gentrified neighbourhoods were mixed (in Montreal adults spent less time being physically active in gentrified neighbourhoods compared to Vancouver where adults spent more time being physically active in gentrified neighbourhoods). The home DA had by far the strongest association with physical activity; on average, participants spent between 2 h and 4 min more per day (Montreal) to 3 h and 54 min more per day (Saskatoon) being physically active in the DA where they lived compared to the amount of time they spent being physically active in other DAs each day (results based on predicted margins of multivariable models, data not shown). The majority of activity happens near or in the home. Forty five percent of time spent being physically active occurred in the same DA where each participants lived. Participant gender and income were also associated with minutes of physical activity. In Vancouver, men recorded fewer minutes of physical activity than women. Households with an annual income of $100,000 + were more physical active than households who made under $50,000. In Vancouver and Victoria, households with an annual income of $100,000 + recorded less physical activity than households with less than $50,000.

### Associations with moderate or vigorous physical activity

Results for time spent in moderate and vigorous physical activity (MVPA) models differed from the previous total physical activity models in both the magnitude and direction of association with environmental characteristics. Each quintile increase in CAN-ALE was associated with 17% (Vancouver) to 57% (Victoria) more minutes of MVPA per DA each day. Patterns within gentrified neighbourhoods were less consistent (Table 3). Participants in Saskatoon and Vancouver spent more minutes in MVPA in gentrified neighbourhoods compared to high socio-economic neighbourhoods, and Vancouver participants also recorded more minutes in MVPA in low socio-economic neighbourhoods, that had not gentrified, relative to high socio-economic areas. The relationships between proximity to amenities and minutes of MVPA were city specific. For instance, minutes of MVPA were negatively associated with proximity to grocery stores in Montreal and Saskatoon but positively associated with grocery stores in Vancouver and Victoria. Proximity to employment was positively associated with MVPA in Montreal but negatively associated with MVPA in the other cities, and proximity to parks was positively associated with MVPA in all cities except Victoria. MVPA was strongly associated with the DA where a participant lived. Seventeen percent of all MVPA minutes were in the same DA where a participant lived (data not shown). Participant gender was only associated with MVPA in Vancouver and income was only associated with MVPA in Saskatoon.

**Table 3:** Associations between built environment and gentrification variables and moderate to vigorous physical activity, by city^a^

	**Montreal**	**Saskatoon**	**Vancouver**	**Victoria**
**Number of people**	**157**	**78**	**150**	**152**
**Can-ALE (quintiles)**	**1.46 (1.37–1.55)**	**1.38 (1.19–1.59)**	**1.17 (1.11–1.23)**	**1.57 (1.47–1.67)**
**Gentrification (Ding)**				
High SES	Reference	Reference	Reference	Reference
Low SES	0.99 (0.91–1.07)	**0.59 (0.49–0.72)**	**1.35 (1.24–1.47)**	**0.78 (0.69–0.88)**
Gentrified	1.04 (0.96–1.13)	**1.47 (1.2–1.81)**	**1.25 (1.16–1.35)**	0.95 (0.84–1.07)
**Urban Compactness**	**0.59 (0.56–0.64)**	**0.74 (0.58–0.93)**	**0.48 (0.44–0.53)**	**0.37 (0.32–0.43)**
**Proximity (quintiles):**				
Employment	**1.07 (1.02–1.13)**	**0.76 (0.63–0.91)**	**0.87 (0.83–0.92)**	**0.65 (0.59–0.71)**
Pharmacy	**1.11 (1.06–1.17)**	1.00 (0.9–1.11)	**1.13 (1.07–1.18)**	**1.16 (1.1–1.23)**
Childcare	**0.92 (0.88–0.96)**	0.97 (0.89–1.06)	**0.81 (0.77–0.84)**	**0.83 (0.79–0.87)**
Health	0.98 (0.94–1.02)	**0.75 (0.67–0.85)**	**1.34 (1.26–1.41)**	0.96 (0.91–1.02)
Grocery	**0.92 (0.88–0.96)**	**0.73 (0.68–0.79)**	**1.06 (1.02–1.1)**	**1.12 (1.07–1.17)**
Primary education	**0.93 (0.9–0.96)**	**0.77 (0.72–0.83)**	**0.86 (0.83–0.9)**	**1.10 (1.05–1.14)**
Secondary education	**0.95 (0.93–0.98)**	**0.89 (0.84–0.95)**	**0.84 (0.81–0.88)**	**0.77 (0.75–0.8)**
Library	0.99 (0.97–1.01)	**1.10 (1.04–1.17)**	**1.10 (1.07–1.12)**	**1.10 (1.07–1.14)**
Transit	0.98 (0.95–1.02)	**1.22 (1.11–1.35)**	**0.83 (0.8–0.86)**	1.00 (0.95–1.06)
Parks	**1.07 (1.04–1.1)**	**1.17 (1.09–1.25)**	**1.16 (1.12–1.19)**	0.98 (0.94–1.02)
**Gender**				
Woman	Reference	Reference	Reference	Reference
Man	0.8 (0.48–1.31)	1.21 (0.81–1.81)	**0.67 (0.47–0.95)**	1.26 (0.96–1.64)
Non-binary	0.92 (0.58–1.46)	**8.63 (1.61–46.22)**		1.03 (0.4–2.68)
**Income groups**				
< $50,000	Reference	Reference	Reference	Reference
$50,000-$99,999	0.80 (0.48–1.31)	1.24 (0.77–2.00)	0.72 (0.42–1.22)	0.95 (0.64–1.4)
$100,000 +	0.92 (0.58–1.46)	**1.45 (0.95–2.21)**	0.76 (0.48–1.22)	0.99 (0.68–1.44)
**Race**				
White	Reference	Reference	Reference	Reference
Visible minority or Indigenous	1.21 (0.63–2.34)	0.83 (0.52–1.32)	0.79 (0.49–1.27)	0.76 (0.48–1.22)
**Age**	1.04 (0.81–1.33)	0.90 (0.68–1.20)	0.79 (0.62–1.00)	**0.82 (0.68–0.98)**
**Home DA**	**9.11 (8.03–10.34)**	**6.64 (5.08–8.69)**	**7.65 (6.61–8.84)**	**11.23 (9.68–13.04)**
**Weekend**	**0.89 (0.84–0.95)**	1.02 (0.90–1.14)	**1.11 (1.04–1.18)**	0.96 (0.90–1.03)
**Precipitation (mm)**	1.00 (1.00–1.01)	0.95 (0.90–1.01)	**0.99 (0.98–1.00)**	1.00 (1.00–1.01)
**Temperature (C)**	**0.99 (0.98–1)**	1.01 (1.00–1.02)	1.01 (0.99–1.03)	0.99 (0.97–1.01)

^a^Results for each model coefficient are reported as incidence rate ratios and 95% confidence intervals

Bold results indicate statistically significant results (*p*-value < 0.05)

### Sensitivity analysis: physical activity and MVPA models in DAs where participants spent more than 5 min per day

Within DAs where participants spent at least five minutes per day, the associations between built environment and gentrification characteristics and time spent being PA or MVPA were attenuated (Additional file 2: Supplement B). By removing observations where less than 5 min were spent in a DA, the average amount of time in each DA each day increased to 36 min in Montreal to 1 h and 29 min in Saskatoon. Results from our multivariable analysis found that for Can-ALE, a one-quintile increase corresponded with 5% to 10% more minutes of PA in a DA each day in Montreal, Vancouver, and Victoria, but Can-ALE was negatively associated with minutes of PA in Saskatoon. Participants in Saskatoon spent more time being PA in high urban compactness areas which is the inverse relationship observed in the other three cities.

## Discussion

The purpose of this study was to examine associations between multiple built environment and gentrification characteristics and physical activity outcomes in four Canadian cities. Our findings provide important methodological contributions to the literature in two important ways. First, we used high spatial resolution exposures that changed when participants move through space. Our analytic approach goes beyond current methods to assess exposure to neighbourhood characteristics by using accelerometer data to document ‘where’ people spend their time and ‘dose’ of how much time they spend in different environments. Second, we used multiple measures of social and built environment features. Previous research has calculated built environment exposures to walkability calculated within limited buffer areas around homes or daily path areas at the trip or day level [31, 32]. Taken together, these two contributions provide some insight in refining the uncertain geographic context problem by examining temporal and spatially detailed data and showing that across the four cities, Can-ALE (a measure of walkability), urban compactness, and parks were important features associated with physical activity. These identified factors have been associated with physical activity in numerous other studies. Our results build on previous studies that have found similar associations between physical activity and the built environment and gentrification.

Previous research has shown that the home location, regardless of whether that location is high or low walkability or near parks, is associated with MVPA [33]. Our results were consistent: participants spent 45% of their time being physical active in their home dissemination area. By including home DA in our multivariable analyses, we were able to account for neighbourhood selection, or the type of neighbourhood a participant chooses to live in, which influences the characteristics of their immediate environment. In addition to home location, we found that the strongest environmental correlates of MVPA were Can-ALE—a composite measure of walkability; urban compactness – a measure of sprawl that includes dimensions of density, mixed use, and street connectivity—across all four cities; and proximity to parks was positively associated with MVPA in three cities. Tamura et al. (2019) examined the association between meeting moderate to vigorous physical activity (MVPA) guidelines and five different built environment measures (population density, street density, land use mix, greenness, and walkability index) using data from 142 participants who wore a GPS and accelerometer device for 1–4 days [34]. Their result showed that greenness was positively associated with MVPA while street density and land use mix were negatively associated with MVPA. In a study with over 800 participants in New York City, neighborhood walkability was associated with total weekly moderate physical activity across the interquartile range of walkability [31]. As well, walking or bicycling to and from a park added an additional 4 to 7 min of MVPA per park visit [35]. The relationship between proximity to grocery stores and both PA and MVPA outcomes varied by city. Grocery store proximity was negatively correlated with MVPA in Montreal and Saskatoon but positively correlated with MVPA in Vancouver and Victoria.

Less consistent with the literature are the findings about gentrification, primary and secondary education, and libraries. We found that time spent in MVPA was greater in gentrified neighborhoods in Saskatoon and Vancouver. Our measure of gentrification is an indicator of social environment conditions that measured changes in housing costs and demographics over the past 10 years. It may be that participants in Vancouver and Saskatoon were drawn to gentrified neighbourhoods because these neighborhoods were perceived as desirable and had attributes that were not included in our set of built environment measures. Regardless of the mechanisms that led adults to be physically active in gentrified neighborhoods, it has important ramifications for population health research as gentrification is correlated with both positive and negative health impacts [36]. The negative associations between time spent in PA and proximity to schools and libraries should be explored further. Our analysis did not consider if participants had children and all participants were adults, which could potentially account for some of the observed differences.

Multi-city comparative studies are crucial to understand what aspects of the built environment generalize across cities and what aspects are city specific in physical activity promotion. We stratified our models by city to shed light on how differences in urban planning, geography, and demographic composition may influence the relationships between urban environmental exposures and physical activity. As evident from the maps, participants in Saskatoon and Montreal were more likely to spend time being active in or around downtown, whereas participants in Victoria and Vancouver spent most of their time being active outside of the city. This can be partially attributed to the availability of large parks outside the city limits in Vancouver or Victoria or differences in the behavior patterns of our participants between cities. Across cities, we found positive correlations between Can-ALE and both physical activity outcomes and negative correlations for urban compactness. In addition, less total physical activity occurred in areas near schools or employment, which has implications for designing safe routes to school and work. For local policy makers and city planners, it is critical that local context is central to the design and implementation of evidence-based physical activity interventions to ensure they have their intended impact. For example, implementing multi-use paths near transit centers in Montreal, Vancouver, and Victoria may increase physical activity and public transit use, whereas this intervention may not be needed in Saskatoon as proximity to public transit was positively correlated with MVPA.

### Limitations

The contributions of our study need to be considered within the context of its limitations. We conducted a multi-level cross sectional study on the built environment and gentrification characteristics linked with time spent being physically active. Our study findings are not generalizable to all physical activity experiences among adults in our four study cities because our study sample is not representative of the underlying population in each city. From comparing study participant demographics (Table 1) to city characteristics (Additional file 1: Supplement A), our study population overrepresented women, high income earners, and white people. More research is needed to understand and ameliorate barriers to participation in accelerometer studies for different populations and develop methods to document physical activity patterns among diverse populations [37].

We do not know the purpose of physical activity or if physical activity occurred inside or outside of a building, which can influence movement patterns measured through accelerometer and GPS data and potentially bias the built environment and gentrification associations with PA. Future combined accelerometer and GPS studies can incorporate trip and dwell detection methods or inside/outside building detection methods in order to examine whether urban environmental exposures vary by trips/dwells or being inside or outside of a building. Last, we did not apply wear time criteria, and physical activity that occurred during the data collection period when a SenseDoc was not worn, would be missing from our analysis. These analyses may not represent usual physical for each participant.

## Conclusion

Our study provides insight into built environment and gentrification characteristics that are associated with the amount of time adults spend being physically active. We found that adults spent more time being physically active near their homes, and in environments that were more walkable and near parks and less time in urban compact areas, regardless of where participants lived. Our analysis also highlighted how proximity to different amenities was linked to physical activity across different cities. These findings enhance our understanding of the influence that built environment and gentrification have on physical activity over time and space, and can support policies to increase physical activity.

## Supplementary Information

**Additional file 1: Supplement A Table. **Comparison between city samples and 2016Canadian Census data on selected socio-demographic characteristics.

**Additional file 2: Supplement B Table 1. **Environmental correlatesof time spent in physical activity in dissemination areas where participantsspent 5+ minutes per day, by city*.** Table 2. **Environmental correlates of timespent in moderate or vigorous physical activity in dissemination areas whereparticipants spent 5+ minutes per day, by city*.

## Data Availability

The datasets generated and/or analyzed during the current study are not publicly available because they contain highly detailed individual location data about participants, but are available from the corresponding author on request. Sharing of location data will also require ethics approval from the requesting author’s institution.

## Abbreviations

GPS: Global Positioning System
GIS: Geographic Information Systems
PA: Physical Activity
MVPA: Moderate to Vigorous Physical Activity
DA: Dissemination Area
CT: Census Tract
SES: Socioeconomic Status
