# Supplementary material for: Using combined Global Position System and accelerometer data points to examine how built environments and gentrification are associated with physical activity in four Canadian cities
Source: Int J Behav Nutr Phys Act. 2022 Jul 7;19:78. doi: 10.1186/s12966-022-01306-z (PMC9261044; doi:10.1186/s12966-022-01306-z)
Supplement: Supplementary file 1 — Additional file 1: Supplement A Table. Comparison between city samples and 2016Canadian Census data on selected socio-demographic characteristics. [file 12966_2022_1306_MOESM1_ESM.docx]

Table. Comparison between city samples and 2016 Canadian Census data on selected socio-demographic characteristics.

|  | Victoria n (%) | Victoria Census % | Vancouver n (%) | Vancouver Census % | Saskatoon n (%) | Saskatoon Census % | Montreal n (%) | Montreal Census % |
| --- | --- | --- | --- | --- | --- | --- | --- | --- |
| Demographics |  |  |  |  |  |  |  |  |
| Age |  |  |  |  |  |  |  |  |
| 15-19 years | 0 (0%) | 5.0 | 2 (0.8%) | 5.8 | 4 (0.4%) | 5.8 | 12 (1.3%) | 5.5 |
| 20-29 years | 46 (20%) | 13.2 | 26 (10.4%) | 14.1 | 111 (11.7%) | 16.0 | 183 (19.3%) | 13.2 |
| 30-39 years | 79 (34.3%) | 12.8 | 28 (11.2%) | 14.2 | 73 (7.7%) | 15.4 | 298 (31.5%) | 14.0 |
| 40-49 years | 60 (26.1%) | 12.4 | 47 (18.8%) | 14.3 | 45 (4.8%) | 12.3 | 195 (20.6%) | 13.5 |
| 50-64 years | 26 (11.3%) | 22.4 | 42 (16.7%) | 21.2 | 7 (0.7%) | 18.6 | 108 (11.4%) | 20.5 |
| 65 and older | 19 (8.3%) | 21.1 | 106 (42.2%) | 15.7 | 7 (0.7%) | 12.8 | 150 (15.9%) | 16.4 |
| Gender |  |  |  |  |  |  |  |  |
| Man | 131 (46.6%) | - | 110 (32.9%) | - | 80 (25.3%) | - | 359 (31.1%) | - |
| Woman | 146 (51.9%) | - | 223 (66.8%) | - | 233 (73.7%) | - | 786 (68.6%) | - |
| Trans Man/Woman | 4 (1.4%) | - | 0 (0%) | - | 1 (0.3%) | - | 1 (0.1%) | - |
| Genderqueer | 0 (0%) | - | 1 (0.3%) | - | 2 (0.6%) | - | 6 (0.5%) | - |
| Prefer not to answer | 0 (0%) | - | 0 (0%) | - | 0 (0%) | - | 3 (0.3%) | - |
| Income |  |  |  |  |  |  |  |  |
| $0-$19,999 | 10 (3.6%) | 10.2 | 14 (4.2%) | 11.5 | 71 (22.5%) | 6.9 | 79 (6.8%) | 11.6 |
| $20,000-$49,999 | 36 (12.8%) | 24.5 | 40 (11.9%) | 22.7 | 69 (21.8%) | 21.0 | 215 (18.6%) | 28.3 |
| $50,000-$99,999 | 107 (38.1%) | 33.5 | 77 (23.1%) | 31.2 | 61 (19.3%) | 31.8 | 378 (32.7%) | 33.7 |
| $100,000-$200,000 | 99 (35.2%) | 26.1 | 97 (29.0%) | 26.5 | 56 (17.7%) | 31.6 | 319 (27.6%) | 21.5 |
| $200,000 and greater | 9 (3.2%) | 5.8 | 49 (14.7%) | 8.1 | 8 (2.5%) | 8.6 | 69 (5.9%) | 5.0 |
| Prefer not to answer | 20 (7.1%) |  | 57 (17.1%) |  | 51 (16.1%) |  | 95 (8.2%) |  |
| Education |  |  |  |  |  |  |  |  |
| Primary/Elementary | 0 (0%) | 7.3 | 0 (0%) | 8.0 | 1 (0.4%) | 8.2 | 0 (0%) | 11.3 |
| Secondary | 42 (15.3%) | 24.8 | 28 (14.7%) | 24.4 | 85 (34.3%) | 26.1 | 44 (6.9%) | 18.3 |
| Trade/Technical | 107 (39.1%) | 31.2 | 46 (24.1%) | 26.0 | 49 (19.8%) | 31.2 | 169 (26.7%) | 34.1 |
| University degree | 125 (45.6%) | 33.8 | 117 (61.3%) | 37.5 | 113 (45.6%) | 31.4 | 419 (66.3%) | 31.9 |
| Graduate degree | 0 (0%) | 2.1 | 138 (72.3%) | 2.6 | 58 (23.4%) | 1.3 | 520 (82.3%) | 2.9 |
| Prefer not to answer | 2 (0.73%) |  | 5 (2.6%) |  | 10 (4.0%) |  | 3 (0.5%) |  |
| Racial Groups |  |  |  |  |  |  |  |  |
| White | 251 (86.9%) | 83.6 | 285 (80.9%) | 50.3 | 225 (66.2%) | 81.2 | 1056 (86.7%) | 75.8 |
| Asian | 19 (6.6%) | 10.8 | 34 (9.7%) | 41.3 | 45 (13.2%) | 12.2 | 51 (4.2%) | 6.7 |
| Black | 0 (0%) | 0.9 | 2 (0.6%) | 1.2 | 9 (2.6%) | 1.9 | 13 (1.1%) | 6.6 |
| Latin American | 4 (1.4%) | 0.7 | 10 (2.8%) | 1.4 | 12 (3.5%) | 0.7 | 28 (2.3%) | 2.7 |
| Middle Eastern | 1 (0.3%) | 0.7 | 2 (0.6%) | 2.5 | 11 (3.2%) | 1.2 | 19 (1.6%) | 5.4 |
| Indigenous | 4 (1.4%) | 6.0 | 4 (1.1%) | 3 | 22 (6.5%) | 11.0 | 6 (0.5%) | 2.9 |
| Racial group not included above | 0 (0%) | - | 12 (3.4%) |  | 10 (2.9%) |  | 31 (2.5%) |  |

Notes. *Participants were able to report multiple ethnic identities, therefore the sum of ethnicities exceeds 100% for each city, as each ethnic group represents people who identify alone or in combination with another ethnicity.
