# Supplementary material for: Using combined Global Position System and accelerometer data points to examine how built environments and gentrification are associated with physical activity in four Canadian cities
Source: Int J Behav Nutr Phys Act. 2022 Jul 7;19:78. doi: 10.1186/s12966-022-01306-z (PMC9261044; doi:10.1186/s12966-022-01306-z)
Supplement: Supplementary file 2 — Additional file 2: Supplement B Table 1. Environmental correlatesof time spent in physical activity in dissemination areas where participantsspent 5+ minutes per day, by city*. Table 2. Environmental correlates of timespent in moderate or vigorous physical activity in dissemination areas whereparticipants spent 5+ minutes per day, by city*. [file 12966_2022_1306_MOESM2_ESM.docx]

**Supplemental A: Sensitivity analysis, PA and MVPA models among DAs where participants spent 5+ minutes per day**

**Table 1. Environmental correlates of time spent in physical activity in dissemination areas where participants spent 5+ minutes per day, by city***

|  | **Montreal** | **Saskatoon** | **Vancouver** | **Victoria** |
| --- | --- | --- | --- | --- |
| **Number of people** | 157 | 78 | 150 | 152 |
| **Can-ALE (quintiles)** | **1.07 (1.01–1.14)** | **0.73 (0.64–0.83)** | **1.05 (1–1.09)** | **1.1 (1.05–1.15)** |
| **Gentrification (Ding)** |  |  |  |  |
| High SES | Reference | Reference | Reference | Reference |
| Low SES | **0.87 (0.8–0.95)** | **0.66 (0.56–0.78)** | 1 (0.93–1.08) | 1.01 (0.92–1.11) |
| Gentrified | **0.83 (0.76–0.9)** | 0.89 (0.75–1.06) | **1.1 (1.03–1.17)** | 0.94 (0.85–1.03) |
| **Sprawl** | 0.97 (0.91–1.03) | **1.49 (1.22–1.82)** | 0.8 (0.73–0.87) | **0.86 (0.76–0.97)** |
| **Proximity (quintiles):** |  |  |  |  |
| Employment | 0.97 (0.92–1.02) | 0.97 (0.84–1.13) | **0.95 (0.91–0.99)** | **0.85 (0.79–0.91)** |
| Pharmacy | 1.02 (0.98–1.07) | **0.59 (0.54–0.65)** | **0.93 (0.89–0.97)** | 0.98 (0.94–1.02) |
| Childcare | **1.07 (1.02–1.11)** | **1.11 (1.02–1.2)** | 0.98 (0.95–1.02) | **1.05 (1.01–1.09)** |
| Health | 1.04 (1–1.09) | **1.25 (1.11–1.4)** | 1.02 (0.97–1.08) | **0.94 (0.9–0.98)** |
| Grocery | **0.87 (0.83–0.9)** | **0.94 (0.88–1)** | 1 (0.97–1.03) | 1 (0.97–1.03) |
| Primary education | **0.96 (0.93–0.99)** | **0.88 (0.83–0.94)** | **1.06 (1.02–1.1)** | 1 (0.96–1.03) |
| Secondary education | **1.03 (1–1.06)** | 1 (0.95–1.06) | 0.98 (0.94–1.01) | **0.95 (0.93–0.98)** |
| Library | **0.97 (0.95–0.99)** | **1.12 (1.06–1.18)** | **1.04 (1.02–1.06)** | 1 (0.97–1.02) |
| Transit | **0.92 (0.89–0.96)** | 1.02 (0.93–1.12) | **0.88 (0.85–0.91)** | **1.06 (1.01–1.11)** |
| Parks | 1.01 (0.99–1.04) | **1.24 (1.17–1.32)** | **1.07 (1.05–1.1)** | 0.98 (0.95–1.02) |
| **Gender** |  |  |  |  |
| Female | Reference | Reference | Reference | Reference |
| Male | 0.97 (0.83–1.13) | 0.93 (0.72–1.21) | 0.93 (0.79–1.09) | 0.95 (0.82–1.11) |
| Non-binary | 0.76 (0.39–1.48) | 2.51 (0.91–6.94) |  | 1.16 (0.69–1.96) |
| **Income groups** |  |  |  |  |
| <$50,000 | Reference | Reference | Reference | Reference |
| $50,000-$99,999 | 1.02 (0.81–1.28) | 1.07 (0.78–1.45) | 1.07 (0.85–1.36) | 0.87 (0.7–1.08) |
| $100,000+ | 0.94 (0.76–1.17) | **1.46 (1.12–1.92)** | 1.05 (0.85–1.3) | 0.82 (0.66–1.01) |
| **Race** |  |  |  |  |
| White | Reference | Reference | Reference | Reference |
| Visible minority or Indigenous | 0.88 (0.65–1.19) | 1.07 (0.79–1.46) | 0.92 (0.74–1.15) | 1.1 (0.84–1.44) |
| **Age** | 1 (0.89–1.12) | 1 (0.83–1.2) | 0.9 (0.8–1) | 0.98 (0.88–1.08) |
| **Home DA** | **7.13 (6.63–7.66)** | **4.77 (4.1–5.54)** | **7.5 (7.01–8.03)** | **7.79 (7.28–8.34)** |
| **Weekend** | 1.04 (0.99–1.1) | 1.04 (0.95–1.14) | **1.06 (1.01–1.11)** | **1.06 (1.01–1.11)** |
| **Precipitation (mm)** | 1 (0.99–1) | 0.96 (0.92–1) | 1 (1–1.01) | 1 (1–1.01) |
| **Temp C** | 1 (0.99–1) | 1.01 (1–1.01) | 1.01 (1–1.02) | 1 (0.99–1.01) |

**Results for each model coefficient are reported as incidence rate ratios and 95% confidence intervals. Bold results indicate statistically significant results (p-value <0.05).*

**Table 2. Environmental correlates of time spent in moderate or vigorous physical activity in dissemination areas where participants spent 5+ minutes per day, by city***

|  | **Montreal** | **Saskatoon** | **Vancouver** | **Victoria** |
| --- | --- | --- | --- | --- |
| **Number of people** | 157 | 78 | 150 | 152 |
| **Can-ALE (quintiles)** | **1.07 (1.01–1.14)** | **0.73 (0.64–0.83)** | **1.05 (1–1.09)** | **1.1 (1.05–1.15)** |
| **Gentrification (Ding)** |  |  |  |  |
| High SES | Reference | Reference | Reference | Reference |
| Low SES | **0.87 (0.8–0.95)** | **0.66 (0.56–0.78)** | 1 (0.93–1.08) | 1.01 (0.92–1.11) |
| Gentrified | **0.83 (0.76–0.9)** | 0.89 (0.75–1.06) | **1.1 (1.03–1.17)** | 0.94 (0.85–1.03) |
| **Sprawl** | 0.97 (0.91–1.03) | **1.49 (1.22–1.82)** | 0.8 (0.73–0.87) | **0.86 (0.76–0.97)** |
| **Proximity (quintiles):** |  |  |  |  |
| Employment | 0.97 (0.92–1.02) | 0.97 (0.84–1.13) | **0.95 (0.91–0.99)** | **0.85 (0.79–0.91)** |
| Pharmacy | 1.02 (0.98–1.07) | **0.59 (0.54–0.65)** | **0.93 (0.89–0.97)** | 0.98 (0.94–1.02) |
| Childcare | **1.07 (1.02–1.11)** | **1.11 (1.02–1.2)** | 0.98 (0.95–1.02) | **1.05 (1.01–1.09)** |
| Health | 1.04 (1–1.09) | **1.25 (1.11–1.4)** | 1.02 (0.97–1.08) | **0.94 (0.9–0.98)** |
| Grocery | **0.87 (0.83–0.9)** | **0.94 (0.88–1)** | 1 (0.97–1.03) | 1 (0.97–1.03) |
| Primary education | **0.96 (0.93–0.99)** | **0.88 (0.83–0.94)** | **1.06 (1.02–1.1)** | 1 (0.96–1.03) |
| Secondary education | **1.03 (1–1.06)** | 1 (0.95–1.06) | 0.98 (0.94–1.01) | **0.95 (0.93–0.98)** |
| Library | **0.97 (0.95–0.99)** | **1.12 (1.06–1.18)** | **1.04 (1.02–1.06)** | 1 (0.97–1.02) |
| Transit | **0.92 (0.89–0.96)** | 1.02 (0.93–1.12) | **0.88 (0.85–0.91)** | **1.06 (1.01–1.11)** |
| Parks | 1.01 (0.99–1.04) | **1.24 (1.17–1.32)** | **1.07 (1.05–1.1)** | 0.98 (0.95–1.02) |
| **Gender** |  |  |  |  |
| Female | Reference | Reference | Reference | Reference |
| Male | 0.97 (0.83–1.13) | 0.93 (0.72–1.21) | 0.93 (0.79–1.09) | 0.95 (0.82–1.11) |
| Non-binary | 0.76 (0.39–1.48) | 2.51 (0.91–6.94) |  | 1.16 (0.69–1.96) |
| **Income groups** |  |  |  |  |
| <$50,000 | Reference | Reference | Reference | Reference |
| $50,000-$99,999 | 1.02 (0.81–1.28) | 1.07 (0.78–1.45) | 1.07 (0.85–1.36) | 0.87 (0.7–1.08) |
| $100,000+ | 0.94 (0.76–1.17) | **1.46 (1.12–1.92)** | 1.05 (0.85–1.3) | 0.82 (0.66–1.01) |
| **Race** |  |  |  |  |
| White | Reference | Reference | Reference | Reference |
| Visible minority or Indigenous | 0.88 (0.65–1.19) | 1.07 (0.79–1.46) | 0.92 (0.74–1.15) | 1.1 (0.84–1.44) |
| **Age** | 1 (0.89–1.12) | 1 (0.83–1.2) | 0.9 (0.8–1) | 0.98 (0.88–1.08) |
| **Home DA** | **7.13 (6.63–7.66)** | **4.77 (4.1–5.54)** | **7.5 (7.01–8.03)** | **7.79 (7.28–8.34)** |
| **Weekend** | 1.04 (0.99–1.1) | 1.04 (0.95–1.14) | **1.06 (1.01–1.11)** | **1.06 (1.01–1.11)** |
| **Precipitation (mm)** | 1 (0.99–1) | 0.96 (0.92–1) | 1 (1–1.01) | 1 (1–1.01) |
| **Temp C** | 1 (0.99–1) | 1.01 (1–1.01) | 1.01 (1–1.02) | 1 (0.99–1.01) |

**Results for each model coefficient are reported as incidence rate ratios and 95% confidence intervals. Bold results indicate statistically significant results (p-value <0.05).*
